# The Dilemma of Treating Delirium: the Conundrum of Drug Management

**DOI:** 10.1007/s11864-022-00987-9

**Published:** 2022-05-11

**Authors:** Meera R. Agar, Ingrid Amgarth-Duff

**Affiliations:** 1 IMPACCT Centre (Improving Palliative, Aged and Chronic Care through Clinical Research and Translation), Faculty of Health, University of Technology Sydney, 235 Jones Street, Ultimo, Sydney, 2007 Australia; 2 South West Sydney Clinical School, University of New South Wales, Sydney, Australia; 3 Ingham Institute of Applied Medical Research, Sydney, Australia

**Keywords:** Delirium, Treatment, Antipsychotics, Benzodiazepines, Palliative care

## Abstract

Delirium is a common medical complication in people living with cancer, particularly with more advanced disease. Delirium is associated with significant symptom burden which causes distress and impacts quality of life. As recommended by international guidelines, a high degree of suspicion is needed to ensure delirium is detected early. Attention to collateral history can provide clues to changes in cognition and attention. Non-pharmacological approaches that can be considered essential elements of care are effective in reducing the risk of delirium. Delirium screening using a validated measure is recommended as even expert clinicians can underdiagnose or miss delirium. The diagnostic assessment requires consideration of the cancer diagnosis and comorbidities, in the context of potential reversibility, goals of care, and patient preferences. The gold standard approach based on expert consensus is to institute management for delirium precipitants supported by non-pharmacological essential care, with the support of an interdisciplinary team. Medication management should be used sparingly and for a limited period of time wherever possible for severe perceptual disturbance or agitation which has not improved with non-pharmacological approaches. Clinicians should be familiar with the registered indication for medications and seek informed consent for off-label use. All interventions put in place to manage delirium need to consider net clinical benefit, including harms such as sedation and loss of capacity for meaningful interaction. Clear communication and explanation are needed regularly, with the person with delirium as far as possible and with surrogate decision makers. Delirium can herald a poor prognosis and this needs to be considered and be discussed as appropriate in shared decision-making. Recall after delirium has resolved is common, and opportunity to talk about this experience and the related distress should be offered during the period after recovery.

## Introduction

Delirium is a serious neuropsychiatric disorder in people with cancer, with high prevalence that exponentially increases as the person is closer to end of life [1••]. The impacts of delirium on the person with cancer are multiple. Delirium impacts on quality of life and contributes to symptom burden through a constellation of changes to attention and awareness; cognition (disorientation, memory and language deficits, and perceptual disturbances); and altered psychomotor behaviour (agitation, physical restlessness, reduced activity), mood, and impaired sleep [2]. Delirium may also potentiate symptom burden in a range of other symptoms including pain [3]. Delirium contributes significantly to morbidity, impacting on function and performance status and other medical complications (pressure injury, falls, and aspiration pneumonia), and contributes to ongoing cognitive decline [4, 5••]. In advanced illness, delirium is an independent predictor of mortality and can herald transition into the end-of-life period [6–8]. Altered arousal and inattention may be associated with higher mortality [9].

The understanding of the epidemiology of delirium is incomplete, with more known in those with advanced cancer. Delirium is relatively frequent in those with advanced cancer, affecting approximately one in ten patients presenting to the emergency department. [10] In acute oncology or internal medicine units, the prevalence ranges from 26 to 47% [11]. Based on limited data, it is thought delirium may occur in over one in eleven older adults receiving chemotherapy [12•].

A systematic approach to delirium detection should be instituted in cancer care, with interdisciplinary non-pharmacological approaches and risk reduction to prevent delirium placed first as much as possible. The most effective approach to manage delirium is to treat the underlying medical precipitants where this is likely to be fruitful, and when it is aligned with the person’s preferences and goals of care. The cause of distress is often multi-factorial, and clinicians should keep an open mind as to the differential diagnoses, as these may require tailored management. It is important that patients and families understand what delirium is, what the causes are, and the management plan. They must be provided with an opportunity to discuss the experience and what is causing the distress. Medication management should be carefully considered with senior clinician support when specific refractory symptoms are present and when medication is instituted at the lowest dose, for the shortest duration required, with regular review.

## Treatment

### Treatment of the underlying cause of delirium

Delirium is potentially reversible, and in advanced cancer, this can be in up to 50% of cases [8, 13, 14, 15]. Clinicians should maintain a high degree of vigilance to ensure early detection of delirium. It is recommended that routine screening is implemented for high-risk patients [11, 16••]. The choice of optimal screening measure should consider the setting, the cancer population, and clinician characteristics including training and skills [17]. A single question (“Do you feel (…the patient’s name) has been more confused lately?”) offers reasonable specificity (87%, 95% CI 74–96) but lower sensitivity (44%, 95% CI 41–80), and is a simple approach that can be integrated into routine clinical histories, adding to the sources of information that may alert the clinical team to the presence of delirium [18].

A thorough clinical assessment is needed to ascertain risk factors and potential delirium precipitants. This includes a full clinical history including collateral history from carers and family, physical and neurological examination, vital signs assessments, and tailored pathology and imaging tests [11]. The potential for reversibility, cancer trajectory, and the person’s preferences, values, and goals of care should be considered [19]. It is likely in most cases that multiple precipitants will be found and will require management consideration, with one study finding between one and six precipitating factors in people with advanced cancer [13].

### Non-pharmacological interventions

Non-pharmacological interventions addressing multiple risk factors have been shown to be effective in reducing the incidence of delirium [20, 21, 22••]. The intervention elements include promoting mobility, nutrition, and hydration; maintaining sensory inputs of vision and hearing; and promoting a normal sleep–wake cycle, regular orientation, and cognitive engagement [20, 21, 22••]. Pain control, optimal bowel and bladder function (avoiding constipation and urinary retention), and management of hypoxia are also considered modifiable risk factors [16]. These are complex interventions which require a comprehensive approach to implementation [22••] involving the service, clinician, and patient and family. Further research is needed to fully understand how these can be optimally adapted for those with advanced cancer with studies yet to show optimal adherence to the full range of strategies [23••].

All cancer patients at risk of delirium should have a medication review, with benzodiazepines posing the highest risk [24]. Opioids can increase the risk of delirium. This is particularly associated with pethidine, but on the other side, inadequately controlled pain is also associated with an increased risk of delirium [24]. The interaction between pain and delirium is complex and bidirectional [25•]. This highlights the importance of consideration of the pathophysiology of the pain syndrome in the cancer patient, tailoring the pain management strategy accordingly with the lowest effective dose [16••] taking into account other physiological factors such as renal and hepatic function. Regular ongoing review of all symptom management medications which have potential for psychoactive side effects is important, including benzodiazepines, opioids, anticholinergics, antidepressants, corticosteroids, and anticonvulsants [19]. Changes in pharmacokinetics or pharmacodynamics when new medications are introduced or dosages changed or ceased are important medication events which can precipitate delirium [16••].

The evidence supporting non-pharmacological measures is stronger for prevention [21] and less conclusive in relation to reducing the duration of delirium. Prevention may merge with treatment when non-pharmacological approaches are in place [16••]. However, two studies of multi-component delirium management including early detection with screening, medication review, and optimising hydration, orientation, and mobilisation did demonstrate earlier alleviation of delirium symptoms, but the studies were of low to moderate quality and did not assess these interventions independent of antipsychotic management [26, 27, 28]. Important principles are as follows: to communicate regularly with the patient and carers, provide information about the diagnosis and ongoing support, provide a supportive environment which reduces noise and fosters orientation, and optimise physiological parameters [16••].

The role of clinically assisted hydration is not fully established and has not been demonstrated to specifically impact delirium symptoms [29]. Hydration can be considered in individual situations where dehydration is deemed to be a significant contributing factor and where oral hydration is not adequate, in pre-renal failure, and to provide fluid maintenance whilst other reversible causes such as infection or hypercalcaemia are being treated [11, 16••]. Infection is a common cause of delirium in people with cancer, and in the presence of sepsis, broad-spectrum antibiotics are recommended until they can be tailored once a causative organism is identified [11]. Bisphosphonates are an effective treatment for hypercalcaemia [11]. If the person is receiving anti-cancer therapies, consideration as to whether these are contributing factors is also important [11]. In the setting of primary or secondary brain tumours, there may be a role for radiotherapy and/or corticosteroids for the management of raised intracranial pressure, but the efficacy of these in reversing delirium has not been established.

### Pharmacological treatment

The pathophysiology of delirium is complex and not fully elucidated, with postulated roles for inflammation, cerebral oxidative metabolism, cortisol and glucose pathways, and aberrant stress responses. The foundation for the common treatment approach of dopamine receptor antagonists is less clear within this context. The Committee for Medicinal Products for Human Use, European Medicines Agency, have approved haloperidol for use in acute delirium when non-pharmacological treatment has not been effective [30], but to our knowledge, there are no other jurisdictions internationally with a registered medication for delirium treatment.

Pharmacological approaches should be carefully considered and they should be used only in refractory situations. In most cases, this is limited to perceptual disturbance or agitation causing significant distress, or if there are safety concerns with risk to the person themselves or others that are not responding to non-pharmacological means [11, 19, 31]. Pharmacological approaches should not be used in isolation and should be seen to supplement non-pharmacological approaches and supporting the person and family with clear information and education [19, 32]. The pros and cons of a pharmacological approach should be discussed with the surrogate decision maker and the person with delirium if able so where possible, a shared decision-making approach to therapy can be achieved [19, 32]. The principles of “start low, go slow” in terms of dose and dose titration can ensure the minimisation of adverse effects. When considering “as required” prescribing, clear parameters must be communicated to nursing clinicians. Bedside nurses are active partners in delirium care and are critical partners in the decision-making for multi-component delirium care, including when “as needed” doses may be warranted.

Before instituting medication management, clinicians should consider the causes and degree of distress, and the potential differential diagnoses. As discussed above, it is possible that physical discomforts such as pain, urinary retention or constipation, inability to find a comfortable position in the bed, or anxiety or fear could be the drivers of distress, and management to address these would be more appropriate and beneficial [19].

#### Antipsychotics

Despite the wide use of antipsychotic medication for delirium, there is no definitive evidence that this reduces delirium duration or severity in hospitalised older adults [33]. A Cochrane review aimed to assess the efficacy of antipsychotics versus non-antipsychotics or placebo primarily on duration of delirium, but also on delirium severity, quality of life, and adverse effects [34]. This review found that antipsychotics did not reduce delirium severity compared to non-antipsychotic medications (standard mean difference (SMD) −1.08, 95% CI −2.55 to 0.39; four studies; 494 participants) [34]. There was also no difference between typical and atypical antipsychotics (SMD −0.17, 95% CI −0.37 to 0.02; seven studies; 542 participants) [34]. The review also found no evidence that antipsychotics resolved delirium symptoms compared to non-antipsychotic drug regimens (relative risk (RR) 0.95, 95% CI 0.30 to 2.98; three studies; 247 participants) and no difference between typical and atypical antipsychotics (RR 1.10, 95% CI 0.79 to 1.52; five studies; 349 participants) [34]. The Cochrane review found overall adverse events to be low (but this is limited by poor reporting in many of the included trials), and no difference in extrapyramidal side effects between typical and atypical antipsychotics [34]. In the intensive care unit setting, they do not reduce delirium duration or coma, survival, and length of intensive care unit or hospital stay [35].

Two randomised placebo-controlled trials have specifically explored symptom control in delirium in palliative care settings. One study found greater delirium symptoms (inappropriate communication, inappropriate behaviour, or perceptual disturbance) in participants who received oral haloperidol and risperidone in comparison to placebo after 72 hours of treatment [36]. This has led to guideline recommendations that haloperidol and risperidone are not indicated in the symptomatic management of mild to moderate delirium and offer no symptomatic benefit [11]. The second study was conducted in advanced cancer patients with delirium in the last days of life, experiencing agitation despite scheduled haloperidol [37]. This study showed reduction in agitation (with associated sedation) with the addition of a single dose of intravenous lorazepam (3 mg) to scheduled haloperidol (2 mg) compared with placebo after 8 hours [37]. A smaller study compared haloperidol in escalating doses, chlorpromazine, or combination therapy in people with advanced cancer and refractory agitation despite low-dose haloperidol in the context of terminal delirium, and found that the three strategies reduced the Richmond Agitation Sedation Scale scores similarly in both groups, and the scores remained low [38].

It is possible that pharmacological strategies (both antipsychotics and benzodiazepines) are predominantly acting by causing sedation, and this can be significant and potentially irreversible. When considering whether pharmacological therapy will offer an improvement in overall symptom burden, it is worth remembering that they may convert a hyperactive delirium to a hypoactive delirium, presenting a new set of distressing symptoms such as drowsiness, lethargy, reduced meaningful communication, and increased inattention [19].

In the setting where pharmacological treatment is deemed necessary, haloperidol will be the first-line choice in most instances. Other first-generation or second-generation antipsychotics are usually considered in the situation where extrapyramidal side effects are of concern or where more sedation may be desired.

##### First-generation antipsychotics

Haloperidol may cause extrapyramidal side effects (EPSEs) and should not be used in people with Parkinson’s disease or dementia with Lewy bodies. It can also cause prolongation of the QTc interval. Starting doses commence at 0.25–0.5 mg as a single dose given orally or subcutaneously and can be given 8 to 12 hourly if regular doses are needed. The dose should be titrated gradually.

Methotrimeprazine (levomepromazine) is significantly more sedating, has anticholinergic effects, and can cause postural hypotension, EPSEs, and paradoxical agitation. Doses commence at 3.25–12.5 mg as a single dose orally or subcutaneously and can be given 8 to 12 hourly if regular doses are needed, with gradual titration of doses [11].

Chlorpromazine is also sedating, has anticholinergic effects, can cause postural hypotension and EPSEs, and can also prolong the QTc interval [11]. Doses commence at 12.5 mg as a single dose orally or subcutaneously and can be given 6–12 hourly if regular doses are needed. The dose should be titrated gradually [11].

##### Second-generation antipsychotics

Olanzapine, risperidone, and quetiapine are less likely to cause EPSEs and vary on the degree in which they cause sedation and postural hypotension [11]. Olanzapine and risperidone are available as oral disintegrating tablets [11], which can be useful when administering to a distressed and agitated patient. Similar to the recommendation for first-generation antipsychotics, starting with a single dose at the lowest dose with slow titration is recommended.

Of note, a US FDA black box warning exists related to mortality associated with antipsychotics when they are used in older people with dementia to manage behavioural disturbances [39].

#### Benzodiazepines

Benzodiazepines are the treatment of choice only for delirium due to benzodiazepine or alcohol withdrawal [11]. They may play a role when sedation is deemed beneficial and to reduce anxiety in a severely distressed patient particularly if the delirium is likely irreversible [11]. The choice of benzodiazepine will be guided by the route of administration and duration of action, depending on whether a short or longer duration of effect is required for the clinical situation.

### Emerging therapies

#### Melatonin and melatonin receptor antagonists

Melatonin, a pineal gland hormone, has been predominantly explored as a preventative pharmacological approach [40]. There is potential that it may also have preventative benefits in advanced cancer patients but this needs further confirmation in larger trials [41]. There is at present insufficient evidence to support its use in clinical practice. Its role in delirium treatment is unknown.

#### Dexmedetomidine

Dexmedetomidine is a highly selective alpha-2 adrenoreceptor agonist. It has the advantage of being able to provide rousable sedation with minimal respiratory depression, analgesic effects, and potential for reduced incidence of delirium in the critical care and perioperative settings. It remains unclear if this is because it allows for the reduction or avoidance of other psychoactive drugs which are deliriogenic [16••].

There is interest in the impact of perioperative factors, including anaesthetic and analgesic management [42], during primary cancer surgery on subsequent cancer recurrence or metastases. The potential of dexmedetomidine to promote cancer metastasis in animal models warrants further exploration [43].

One open-label study in the palliative care setting [44] where titrated dexmedetomidine was used for hyperactive delirium at the end of life in 22 participants demonstrated a reduction in delirium severity. Interestingly, 50% of participants crossed over to standard care, with the predominant reason being a desire for deeper sedation.

### Paediatric considerations

There is limited data on the incidence of delirium in childhood cancer, but one study reports an incidence of 18.8% [45]. In paediatrics, hypoactive presentations are common, and diagnosis may require more attention to behavioural changes rather than the cognitive features in adults. Similar to adults, early identification is critical, and regular screening using instruments suitable for the paediatric population is recommended [46]. Treatment relies on management of the underlying cause and providing a supportive environment [46]. Benzodiazepines should be avoided in children as in adults, as they can prolong delirium and agitation [46]. Haloperidol, risperidone, olanzapine, and quetiapine have all been studied in delirium in children [46]. There is also emerging interest in the role of dexmedetomidine in paediatric intensive care [46].

## Summary

Delirium is a complex medical emergency causing significant symptom burden. Optimal management occurs in health systems that implement approaches to detect delirium early and support interdisciplinary non-pharmacological care. When pharmacological management is indicated, it requires shared decision-making and consideration of net clinical benefit under the supervision of a senior clinician working closely with the full clinical team. Further research is needed to identify more optimal pharmacological therapies.
